# O Agonismo do GPR84 Atenua a Inflamação Endotelial Induzida por Ácidos Graxos Livres e a Adesão de Monócitos através da Via Gfi1/NF-Κb

**DOI:** 10.36660/abc.20260231

**Published:** 2026-09-10

**Authors:** Li Li, Gang Fu, Yanling Liu, Caiyun Liu

**Affiliations:** 1 Department of Cardiology The Third People’s Hospital of Longgang District Shenzhen Guangdong Province China Department of Cardiology, The Third People’s Hospital of Longgang District Shenzhen, Guangdong Province – China

**Keywords:** Aterosclerose, Endotélio Vascular, Monócitos

## Abstract

**Fundamento:**

A disfunção endotelial e a adesão de monócitos induzidas por ácidos graxos livres (AGLs) são eventos iniciais críticos na aterosclerose. O palmitato, um ácido graxo livre saturado de cadeia longa, aumentou a expressão de GPR84. Embora o GPR84 seja canonicamente ativado por AGLs de cadeia média, sua indução sob estresse lipotóxico sugere um papel compensatório e dependente do contexto. Seu papel na inflamação endotelial ainda não está bem definido.

**Objetivos:**

Investigar o papel funcional da ativação do GPR84 na inflamação endotelial induzida por AGLs e elucidar os mecanismos subjacentes.

**Métodos:**

Células endoteliais da aorta humana (HAECs) foram desafiadas com palmitato (0,5 ou 1 mM) na presença ou ausência dos agonistas de GPR84, 6-OAU ou ZQ-16 (cada um a 1 μM). A expressão de citocinas inflamatórias e moléculas de adesão foi avaliada por qPCR e ELISA. A adesão de monócitos foi avaliada utilizando células THP-1. A translocação nuclear de NF-κB p65 e a expressão de fator de crescimento independente 1 (Gfi1) foram examinadas, e o silenciamento de Gfi1 foi realizado para avaliar a relevância funcional.

**Resultados:**

O palmitato aumentou a expressão de GPR84 em HAECs. O tratamento com agonistas de GPR84 reduziu significativamente a superexpressão induzida por palmitato de IL-1β, TNF-α, MCP-1, VCAM-1 e ICAM-1, tanto em nível de mRNA quanto de proteína, e atenuou a adesão de monócitos THP-1. Mecanisticamente, a ativação de GPR84 inibiu a translocação nuclear de NF-κB p65 e preservou a expressão de Gfi1. O silenciamento de Gfi1 anulou os efeitos protetores dos agonistas de GPR84, restaurando a ativação de NF-κB, a expressão de moléculas de adesão e a adesão de monócitos.

**Conclusões:**

O agonismo do GPR84 atenua a inflamação endotelial induzida por AGLs ao restaurar a expressão de Gfi1 e suprimir a sinalização de NF-κB, destacando uma potencial estratégia terapêutica para aterosclerose precoce que justifica validação in vivo adicional.

## Introdução

As patologias cardiovasculares subjacentes ao infarto do miocárdio, acidente vascular cerebral e doença arterial periférica representam coletivamente a principal causa de mortalidade em países desenvolvidos e em desenvolvimento.^1^ As paredes arteriais são revestidas por uma única camada de células endoteliais metabolicamente ativas que coordenam a função vasomotora, a adesão de leucócitos e as cascatas inflamatórias para preservar a homeostase vascular.^2^ Alterações metabólicas associadas à obesidade e ao diabetes mellitus causam ativação endotelial, convertendo essa barreira protetora em uma superfície pró-inflamatória que atrai leucócitos circulantes.^3^ Estressores oxidativos e lipoproteínas modificadas, incluindo lipoproteína de baixa densidade oxidada (LDL-ox), acumulam-se na íntima subendotelial, causando alterações fenotípicas e aumentando a produção de glicoproteínas adesivas que promovem a ancoragem de monócitos e a subsequente transmigração.^4^

Ácidos graxos livres (AGLs) em quantidades aumentadas em condições de dislipidemia danificam diretamente a integridade endotelial, gerando espécies reativas de oxigênio e ativando receptores de reconhecimento de padrões.^5^ O ácido palmítico, um ácido graxo saturado de dezesseis carbonos presente nos lipídios da dieta, estimula a ativação endotelial, como observado pelo aumento da produção da molécula de adesão celular vascular-1 (VCAM-1) e da molécula de adesão intercelular-1 (ICAM-1).^6^ Em células endoteliais da aorta humana (HAECs), a exposição ao palmitato desencadeia a translocação nuclear do NF-κB e a regulação transcricional positiva de múltiplos mediadores pró-inflamatórios, incluindo IL-1β, TNF-α e MCP-1.^7^ Essas alterações moleculares melhoram a capacidade adesiva endotelial, o que promove a adesão e a diapedese de monócitos circulantes na parede arterial.^8^

A adesão de monócitos às superfícies endoteliais ativas é uma etapa limitante importante na formação de ateroma.^9^ Após forte adesão, os monócitos atravessam a barreira endotelial e se desenvolvem em macrófagos na camada íntima, onde ingerem lipoproteínas oxidadas e se convertem em células espumosas ricas em colesterol.^10^ A família de fatores de transcrição do fator nuclear kappa B coordena a ativação endotelial regulando a produção de moléculas de adesão e mediadores inflamatórios solúveis em resposta a citocinas ou padrões moleculares derivados de lipídios.^11^ A sinalização canônica do fator nuclear kappa B inclui a fosforilação de proteínas inibidoras, a translocação nuclear de heterodímeros p65-p50 e a ligação ao promotor em sítios consenso kappa B encontrados em muitos genes pró-inflamatórios.^12^ Mecanismos homeostáticos de feedback negativo normalmente interrompem as reações inflamatórias, mas o estresse metabólico prolongado parece perturbar esses sistemas regulatórios, levando à ativação endotelial persistente e danos vasculares progressivos.^13^

O receptor acoplado à proteína G 84 (GPR84) foi identificado como um possível regulador de respostas inflamatórias por meio da ativação seletiva por AGLs de cadeia média.^14^ O GPR84 foi inicialmente identificado como um receptor órfão expresso apenas em linhagens mieloides, mas investigações estruturais subsequentes mostram que ele se liga a proteínas Gi/o heterotriméricas, reduzindo as concentrações intracelulares de adenosina monofosfato cíclico após a ligação do agonista.^15^ Estudos farmacológicos demonstram que agonistas sintéticos, como o 6-OAU e o ZQ-16, influenciam a polarização inflamatória de macrófagos e as atividades efetoras de neutrófilos.^16^ Estudos utilizando modelos de deleção genética mostram que esse receptor desempenha um papel na cicatrização de feridas, na inflamação da mucosa e na dor neuropática, embora sua função no endotélio vascular ainda seja pouco compreendida.^17^ Os padrões de expressão e as ramificações funcionais da ativação do GPR84 em HAECs tratadas com ácidos graxos saturados não foram completamente investigados. Embora o palmitato esteja fora da faixa típica de ligação de cadeia média do GPR84, sabe-se que o GPR84 modula as respostas celulares ao estresse lipotóxico induzido por ácidos graxos saturados de cadeia longa.^18^

O fator de crescimento independente 1 (Gfi1) é um repressor transcricional com dedos de zinco que regula as respostas imunes inatas e a diferenciação hematopoiética.^19^ Pesquisas anteriores demonstraram que o Gfi1 reduz a atividade do fator nuclear κB ao bloquear a ligação do p65 ao DNA nos promotores dos genes-alvo, reduzindo a produção de citocinas inflamatórias.^20^ A ativação do receptor *Toll-like* induz a expressão de Gfi1, que faz parte de um circuito de retroalimentação negativa que limita reações inflamatórias excessivas.^21^ Estudos recentes mostram que a perda de Gfi1 em macrófagos causa ativação inflamatória espontânea caracterizada pelo aumento da sinalização do fator nuclear κB e liberação de citocinas.^22^ Embora Gfi1 tenha sido extensivamente pesquisado em linhagens hematológicas, sua expressão e envolvimento funcional em células endoteliais submetidas a estresse metabólico são menos compreendidos.

Nossa hipótese era de que a ativação do GPR84 influenciaria a inflamação endotelial induzida pelo ácido palmítico, regulando a expressão de Gfi1 e inibindo a sinalização do fator nuclear κB (NF-κB). Este estudo demonstra que o ácido palmítico estimula a expressão de GPR84 em HAECs; entretanto, o agonismo farmacológico do GPR84 reduz a produção de citocinas inflamatórias, a expressão de moléculas de adesão e a adesão de monócitos. Investigações mecanísticas mostram que a ativação do GPR84 mantém a expressão de Gfi1 em condições inflamatórias, mas a deleção genética de Gfi1 reverte os benefícios protetores do agonismo do receptor. Esses resultados estabelecem um novo eixo GPR84-Gfi1-NF-κB que modula as respostas inflamatórias endoteliais e pode representar um alvo terapêutico para a proteção vascular em doenças metabólicas.

## Materiais e métodos

### Reagentes e anticorpos

O ácido palmítico (ácido livre, P0500), a albumina sérica bovina livre de ácidos graxos (A8806) e todos os reagentes químicos de uso geral para laboratório foram adquiridos da Sigma-Aldrich, salvo indicação em contrário. O agonista sintético de GPR84, 6-OAU (13583), foi obtido da Cayman Chemical, e o agonista de GPR84, ZQ-16 (HY-112042), foi obtido da MedChemExpress. Os seguintes anticorpos primários foram utilizados para a imunodetecção: anticorpo monoclonal de camundongo anti-GPR84 (Santa Cruz Biotechnology, sc-514951), anticorpos monoclonais de coelho contra NF-κB p65 (Cell Signaling Technology, 8242), Gfi1 total (Cell Signaling Technology, 31929) e Lamin B1 (Cell Signaling Technology, 13435), juntamente com o anticorpo policlonal de coelho contra β-actina (Cell Signaling Technology, 4967). Os anticorpos secundários conjugados à peroxidase de rábano incluíram IgG anti-coelho (Cell Signaling Technology, 7074) e IgG anti-camundongo (Cell Signaling Technology, 7076). Sistemas comerciais de ELISA para quantificação de IL-1β humana (DY201), TNF-α (DY210), MCP-1 (DY279), VCAM-1 (DY809) e ICAM-1 (DY720) foram adquiridos da R&D Systems. Vetores adenovirais codificando shRNA direcionado a Gfi1 (sc-35467-V) e shRNA controle aleatório (sc-108080) foram fornecidos pela Santa Cruz Biotechnology.

### Cultura e manutenção de células

Células endoteliais aórticas humanas primárias (Lonza, CC-2535) foram cultivadas em meio EGM-2 (Lonza, CC-3162) suplementado com os fatores de crescimento fornecidos pelo fabricante, incluindo 2% de FBS, hidrocortisona, hFGF, VEGF, R3-IGF-1, ácido ascórbico, hEGF e GA-1000. As condições de incubação foram mantidas a 37 °C com 5% de CO_2_ em câmara úmida, e as células foram utilizadas até a sexta passagem. Monócitos THP-1 (ATCC, TIB-202) foram mantidos em RPMI 1640 (Gibco, 11875093) contendo 10% de FBS, 2 mM de L-glutamina, 100 U/mL de penicilina e 100 μg/mL de estreptomicina. Para evitar a diferenciação espontânea, a densidade celular foi mantida entre 2×10^5^ e 1×10^6^ células/mL durante todo o cultivo. Todas as linhagens celulares foram testadas para contaminação por micoplasma utilizando o kit de detecção de micoplasma MycoAlert (Lonza, LT07-218) no início da passagem e a cada quatro semanas subsequentes; todas as culturas utilizadas neste estudo apresentaram resultado negativo.

### Preparação de complexos de ácidos graxos-albumina

Para preparar o ácido palmítico para experimentos de cultura celular, primeiro dissolvemos o ácido livre em NaOH 0,1 M (esterilizado por filtração) a 70 °C sob agitação constante até a clarificação da solução. Após o resfriamento, o pH foi ajustado para 7,4 com HCl 1 M, e a solução estoque resultante foi filtrada através de um filtro de 0,22 μm para garantir a esterilidade. Para uso experimental, essa solução estoque de 100 mM foi complexada com BSA livre de ácidos graxos em meio basal sem soro na proporção molar de 5:1, resultando em concentrações finais de trabalho de 0,5 mM ou 1 mM de ácido palmítico imediatamente antes do tratamento celular. A concentração final de BSA foi de aproximadamente 0,834% (p/v) na concentração de trabalho de 1 mM de palmitato, correspondendo à proporção molar de 5:1. Os complexos foram utilizados imediatamente após o preparo para evitar a precipitação. Antes da aplicação dos complexos palmitato-BSA, as células foram mantidas por 12 horas em meio contendo 2% de FBS para reduzir a ativação basal, conforme descrito na seção Estimulação Farmacológica.

### Estimulação farmacológica e transdução de shRNA

Antes dos tratamentos experimentais, monocamadas confluentes de HAEC foram submetidas a privação de soro por 12 horas em meio EGM-2 contendo 2% de FBS para minimizar a ativação basal. As células foram então expostas a conjugados de ácido palmítico-BSA (0,5 mM ou 1 mM final) por 24 horas, isoladamente ou concomitantemente com os agonistas de GPR84 6-OAU (1 μM) ou ZQ-16 (1 μM). Para os estudos de perda de função, as HAECs foram incubadas por 48 horas com adenovírus codificando shRNA direcionado a Gfi1 ou sequências de controle aleatórias com uma MOI de 50. A eficiência do *knockdown* foi verificada por análise de Western blot, que mostrou uma redução de aproximadamente 70% nos níveis da proteína Gfi1.

### Extração de RNA e PCR quantitativo em tempo real

Extraímos o RNA total de células cultivadas utilizando o reagente TRIzol (Invitrogen, 15596026), seguindo o protocolo fornecido pelo fabricante. O cDNA de primeira fita foi obtido por transcrição reversa a partir de 1 μg de RNA purificado, utilizando o kit de síntese de cDNA iScript (Bio-Rad, 1708891). As reações de PCR quantitativa foram realizadas com o SYBR Green PCR Master Mix (Applied Biosystems, 4368577) em um instrumento QuantStudio 3 (Applied Biosystems). As condições de termociclagem foram: 95 °C por 10 min, seguidas por 40 ciclos de 95 °C por 15 s e 60 °C por 1 min, com uma etapa final de curva de fusão. As sequências dos primers para os seguintes genes humanos foram obtidas do banco de dados PrimerBank: GPR84 (F: 5’-TTGGCATCTTCTATTGCCTCATC-3’, R: 5’-TGTCGCAACTTGTATTGGTCC-3’); IL-1β (F: 5’-AAACAGATGAAGTGCTCCTTCCAGG-3’, R: 5’-TGGAGAACACCACTTGTTGCTCCA-3’); TNF-α (F: 5’-GAGGCCAAGCCCTGGTATG-3’, R: 5’-CGGGCCGATTGATCTCAGC-3’); MCP-1 (F: 5’-CAGCCAGATGCAATCAATGCC-3’, R: 5’-TGGAATCCTGAACCCACTTCT-3’); VCAM-1 (F: 5’-GGGAAGATGGTCGTGATCCTT-3’, R: 5’-TCTGGGGTGGTCTCGATTTTA-3’); ICAM-1 (F: 5’-ATGCCCAGACATCTGTGTCC-3’, R: 5’-GGGGTCTCTATGCCCAACAA-3’); Gfi1 (F: 5’-CCGCGCTCATTTCTCGTCA-3’, R: 5’-ACGGAGGGAATAGTCTGGTCC-3’). 18S 18S rRNA (F: 5’-GTAACCCGTTGAACCCCATT-3’, R: 5’-CCATCCAATCGGTAGTAGCG-3’) foi usado como referência interna para normalização. A expressão do gene alvo relativa ao controle 18S rRNA foi calculada usando o método de Ct comparativo (2^-^).

### Extração de proteínas e análise por Imunoblot

Preparamos lisados de células inteiras coletando as células em tampão RIPA (Thermo, 89900) contendo um coquetel de inibidores de proteases e fosfatases (Thermo, A32959). As frações nucleares e citoplasmáticas foram separadas utilizando o sistema de extração NE-PER (Thermo, 78833) de acordo com as instruções do fabricante. As concentrações de proteína em cada amostra foram medidas utilizando o ensaio BCA (Pierce, 23225). As amostras de proteína (20 μg por poço) foram separadas por SDS-PAGE em géis de poliacrilamida a 10% e, em seguida, transferidas eletroforeticamente para membranas de PVDF (Millipore, IPVH00010). Para minimizar a ligação não específica, as membranas foram incubadas por 1 hora à temperatura ambiente em solução bloqueadora (5% de leite desnatado em TBST), seguida de incubação durante a noite a 4°C com anticorpos primários diluídos a 1:1000 em solução bloqueadora. Após lavagens extensivas, as membranas foram incubadas por 1 hora à temperatura ambiente com anticorpos secundários conjugados com HRP (diluição 1:2000). A β-actina foi utilizada como controle de carregamento para lisados de células inteiras, enquanto a Lamin B1 serviu como controle de carregamento para frações nucleares. As bandas de proteína foram detectadas utilizando o substrato Pierce ECL (Thermo, 32106) e as intensidades dos sinais foram quantificadas por densitometria com o software ImageJ (NIH).

### Quantificação de citocinas e moléculas de adesão

Medimos as concentrações de proteínas secretadas de IL-1β, TNF-α, MCP-1, VCAM-1 e ICAM-1 nos sobrenadantes de cultura coletados, utilizando kits DuoSet ELISA (R&D Systems) de acordo com os procedimentos recomendados pelo fabricante. As leituras de absorbância foram realizadas a 450 nm, com correção de fundo a 540 nm, em um leitor de microplacas BioTek Synergy H1. As concentrações das amostras foram interpoladas a partir de curvas padrão e, em seguida, normalizadas para o conteúdo total de proteína nos lisados celular

### Ensaio de adesão de monócitos

As células THP-1 foram coletadas, lavadas e ressuspensas em meio RPMI 1640 sem soro, na densidade de 2 × 10^5^ células por mililitro. As suspensões celulares foram incubadas com 1 μM de calceína-AM (Invitrogen C3099) por 30 minutos a 37 °C, com agitação intermitente, seguidas de três lavagens com solução salina tamponada com fosfato para remover o corante não incorporado. As células THP-1 marcadas (2 × 10^5^ células por poço) foram co-incubadas com monocamadas de HAEC tratadas em placas de 24 poços por exatamente uma hora a 37 °C. As células não aderentes foram removidas por aspiração suave do meio, seguida de duas lavagens rápidas com 1 mL de solução salina tamponada com fosfato pré-aquecida (37 °C) por poço. As células aderentes foram visualizadas imediatamente usando um microscópio de fluorescência invertido (excitação 494 nm, emissão 517 nm) equipado com uma objetiva de 10×. Imagens representativas foram capturadas para cada condição experimental. Para a análise quantitativa, as células aderidas foram contadas em cinco campos aleatórios por poço utilizando o software ImageJ (National Institutes of Health) por um investigador que desconhecia os grupos de tratamento, e os resultados foram expressos como variação relativa em comparação com os controles não tratados.

### Ensaio do gene repórter da luciferase

As células HAEC foram plaqueadas na densidade de 1 × 10^5^ células por poço em placas de 24 poços, 24 horas antes da transfecção. As células foram submetidas à transfecção transitória com o plasmídeo repórter de luciferase de vaga-lume NF-κB (pNF-κB-Luc, Promega, Cat. nº E8491) e o vetor controle de luciferase de Renilla (pRL-TK, Promega E2241) utilizando Lipofectamine 3000 (Invitrogen L3000015), seguindo as especificações do fabricante. Após 24 horas de recuperação, as células transfectadas foram submetidas à privação de soro e subsequente tratamento farmacológico, conforme descrito. Os lisados celulares foram preparados utilizando o tampão de lise passiva (Promega E1941). As atividades das luciferases de vaga-lume e de Renilla foram medidas sequencialmente utilizando o sistema de ensaio de repórter de dupla luciferase (Promega E1910) em um luminômetro (Turner BioSystems Modulus). A atividade transcricional do NF-κB foi calculada como a razão entre a atividade da luciferase de vaga-lume e a da luciferase de Renilla, e normalizada em relação ao controle não tratado.

### Análise estatística

Todos os dados quantitativos são expressos como média ± erro padrão da média (EPM), derivados de pelo menos três experimentos independentes realizados em duplicata ou triplicata. Antes da ANOVA, os dados foram avaliados quanto à normalidade pelo teste de Shapiro-Wilk e quanto à homogeneidade das variâncias pelo teste de Levene. Todos os conjuntos de dados atenderam às premissas necessárias para a análise paramétrica. Para determinar a significância estatística, aplicamos ANOVA de uma via seguida por testes post-hoc apropriados (Tukey, Dunnett ou Holm-Šidák) para comparações entre múltiplos grupos, ou ANOVA de duas vias com correção de Šidák para experimentos com duas variáveis independentes. As diferenças foram consideradas estatisticamente significativas para p < 0,05. Todos os cálculos estatísticos e a geração de gráficos foram realizados utilizando o GraphPad Prism versão 9.0 (GraphPad Software, La Jolla, CA).

## Resultados

### Indução de GPR84 pelo ácido palmítico em células endoteliais da aorta humana após exposição de 24 horas

Inicialmente, buscamos determinar se o ácido palmítico, um ácido graxo saturado de cadeia longa pró-inflamatório representativo, modula a expressão de GPR84 após 24 horas de exposição em HAECs sob condições experimentais controladas. A exposição ao ácido palmítico em concentrações de 0,5 mM e 1 mM por 24 horas produziu aumentos dependentes da concentração na abundância do transcrito de GPR84 em relação aos controles não tratados, conforme quantificado por reação em cadeia da polimerase em tempo real (Figura 1A). Análises simultâneas por *Western blot* corroboraram essas alterações transcricionais no nível proteico, demonstrando uma acentuada regulação positiva da imunorreatividade de GPR84 após protocolos de estimulação idênticos (Figura 1B). Esses dados indicam que o ácido palmítico induz a expressão de GPR84 por meio de mecanismos que operam tanto em nível transcricional quanto proteico.

**Figura 1:**
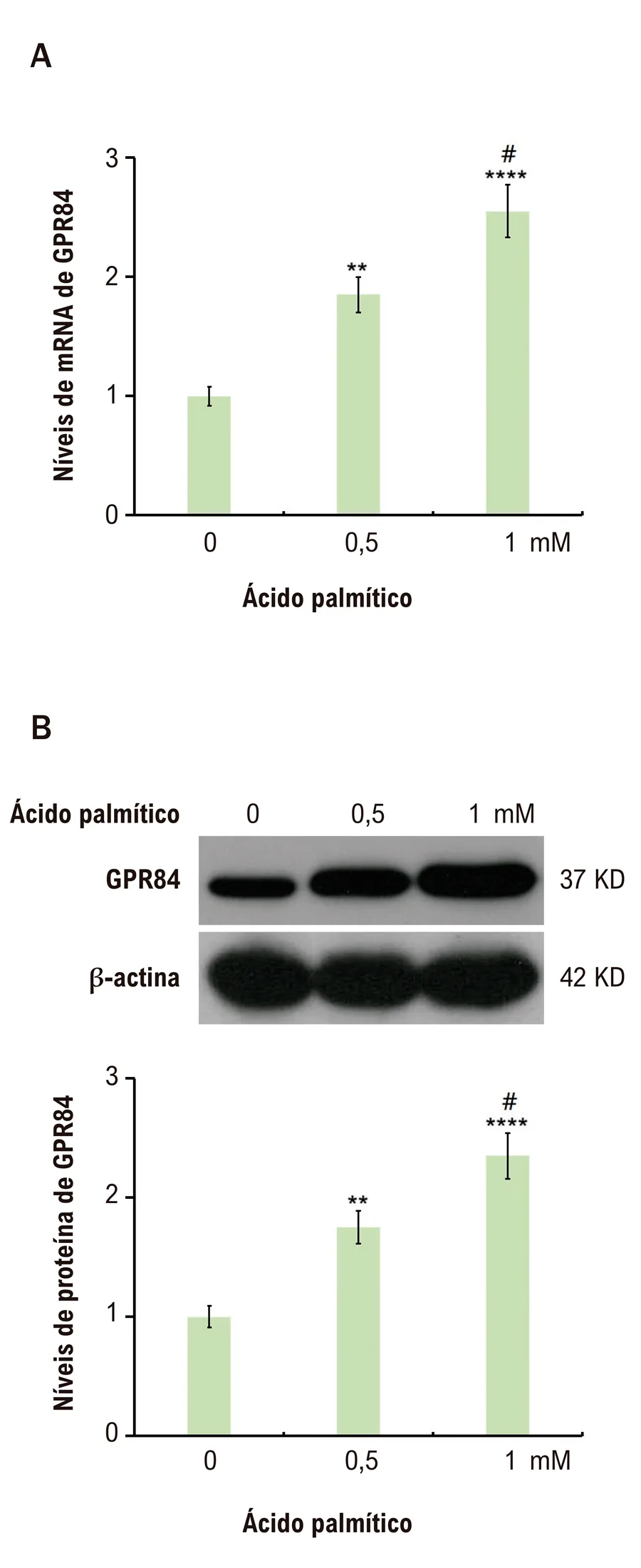
– O ácido palmítico induz a expressão de GPR84 em células endoteliais da aorta humana (HAECs). As células foram tratadas com ácido palmítico a 0,5 ou 1 mM durante 24 horas. (A) PCR quantitativo (qPCR) mostrando os níveis de mRNA de GPR84 normalizados para 18S rRNA. (B) Imunoblotagens representativas e quantificação dos níveis da proteína GPR84 em relação à β-actina. **P < 0,01, ****P < 0,0001 em comparação com os controles não tratados; #P < 0,05 entre os grupos de ácido palmítico a 0,5 mM e 1 mM (ANOVA de uma via com teste post-hoc de Tukey).

### Atenuação da produção de citocinas pró-inflamatórias via agonismo de GPR84

Em seguida, examinamos se a ativação farmacológica do GPR84, utilizando agonistas sintéticos estruturalmente distintos, atenua a cascata de citocinas pró-inflamatórias iniciada pelo desafio com ácido palmítico. Análises quantitativas por PCR em tempo real revelaram que o pré-tratamento com 6-OAU ou ZQ-16 diminuiu substancialmente a regulação positiva do mRNA da interleucina-1β induzida pelo ácido palmítico (Fig. 2A). Padrões inibitórios análogos surgiram para os níveis de transcritos do fator de necrose tumoral-α (Fig. 2B) e para a expressão do mRNA da proteína quimioatraente de monócitos-1 (Fig. 2C). Ensaios imunoenzimáticos (ELISA) realizados em meio de cultura condicionado confirmaram que essas modificações transcricionais se traduziram em reduções correspondentes nas concentrações da proteína IL-1β secretada (Fig. 2D), nos níveis da proteína TNF-α (Fig. 2E) e na abundância da proteína MCP-1 (Fig. 2F). Em conjunto, esses resultados demonstram que o agonismo do GPR84 suprime a produção de mediadores inflamatórios induzida pelo ácido palmítico através da regulação coordenada da expressão gênica e da secreção de proteínas.

**Figura 2:**
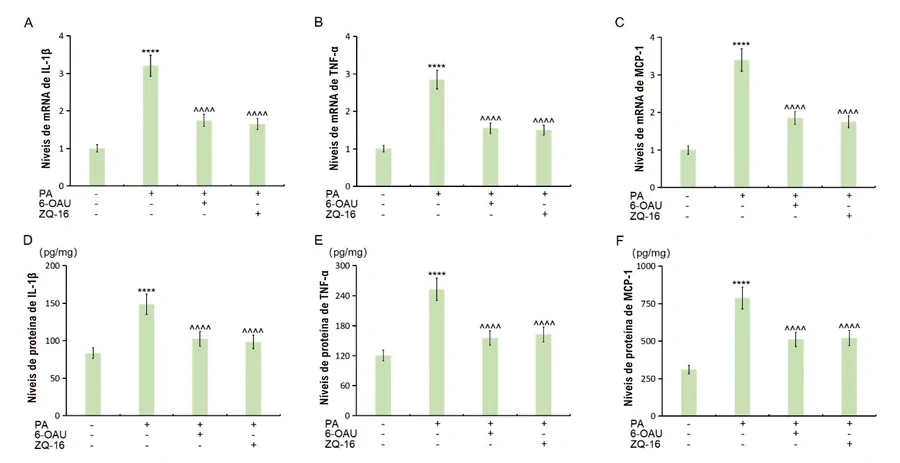
– Os agonistas de GPR84 atenuam a produção de citocinas pró-inflamatórias. Células endoteliais da aorta humana (HAECs) foram estimuladas por 24 horas com 1 mM de ácido palmítico (PA), isoladamente ou em conjunto com 1 μM de 6-n-octilaminouracila (6-OAU) ou 1 μM de ZQ-16. (A–C) Expressão relativa de mRNA de interleucina-1β (IL-1β), fator de necrose tumoral-α (TNF-α) e proteína quimioatraente de monócitos-1 (MCP-1) medida por qPCR. (D–F) As concentrações correspondentes de proteínas secretadas nos sobrenadantes de cultura foram quantificadas por ensaio imunoenzimático (ELISA). ****p < 0,0001 versus controles não tratados; ^^^^p < 0,0001 versus o grupo tratado apenas com PA (ANOVA de uma via com teste post-hoc de Tukey).

### Modulação da expressão de moléculas de adesão endotelial

Considerando o papel central da VCAM-1 e da ICAM-1 no recrutamento de monócitos circulantes para o endotélio ativado, avaliamos subsequentemente se a ativação do GPR84 modula a expressão desses receptores de adesão essenciais. O desafio com ácido palmítico induziu acentuadamente a expressão de mRNA de VCAM-1 acima dos níveis basais, enquanto a co-incubação com 6-OAU ou ZQ-16 atenuou essa resposta transcricional de maneira estatisticamente significativa (Fig. 3A). Condições experimentais paralelas demonstraram reduções comparáveis nos níveis de transcritos de ICAM-1 após o tratamento com o agonista (Fig. 3B). A quantificação de proteínas por meio de ensaios imunoenzimáticos específicos confirmou que a exposição ao agonista diminuiu significativamente a secreção de VCAM-1 nos sobrenadantes de cultura (Fig. 3C), enquanto as concentrações de proteína ICAM-1 mostraram reduções análogas sob condições de tratamento idênticas (Fig. 3D). Em conjunto, esses resultados revelam que a ativação do GPR84 atenua os fenótipos de ativação endotelial por meio da regulação negativa de receptores de adesão essenciais para a adesão de leucócitos.

**Figura 3:**
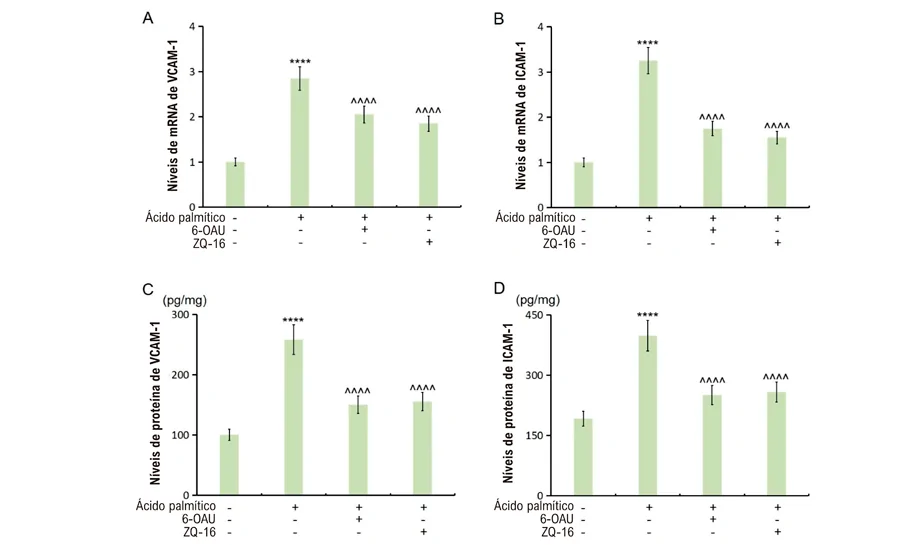
– O tratamento com agonista reduz a expressão da molécula de adesão endotelial. Após 24 horas de exposição a 1 mM de PA na presença ou ausência dos agonistas indicados (1 μM cada), as células endoteliais da aorta humana (HAECs) foram avaliadas quanto à expressão da molécula de adesão celular vascular-1 (VCAM-1) e da molécula de adesão intercelular-1 (ICAM-1). (A, B) Níveis de mRNA determinados por qPCR. (C, D) Concentrações de proteína secretada medidas por ensaio imunoenzimático (ELISA). ****p < 0,0001 versus controles não tratados; ^^^^p < 0,0001 versus o grupo tratado apenas com PA (ANOVA de uma via com teste post-hoc de Tukey).

### Inibição funcional da adesão de monócitos ao endotélio

Para determinar se as alterações moleculares observadas culminam em mudanças funcionais nas interações celulares, avaliamos a adesão de monócitos THP-1 a monocamadas endoteliais tratadas usando metodologias de fluorescência quantitativa. O exame microscópico de monócitos corados com calceína-AM revelou uma adesão pontilhada densa às monocamadas de HAEC tratadas com ácido palmítico, enquanto as culturas submetidas à co-incubação com agonistas de GPR84 apresentaram intensidade de fluorescência marcadamente atenuada, indicando redução da adesão celular (Figura 4A). A análise quantitativa de imagens corroborou essas observações qualitativas, demonstrando que tanto o 6-OAU quanto o ZQ-16 reduziram os índices de adesão de monócitos em aproximadamente cinquenta por cento em relação à estimulação apenas com ácido palmítico (Figura 4B). Essas evidências funcionais indicam que o agonismo de GPR84 interrompe a interface adesiva entre células endoteliais ativadas e monócitos circulantes em condições fisiopatologicamente relevantes.

**Figura 4:**
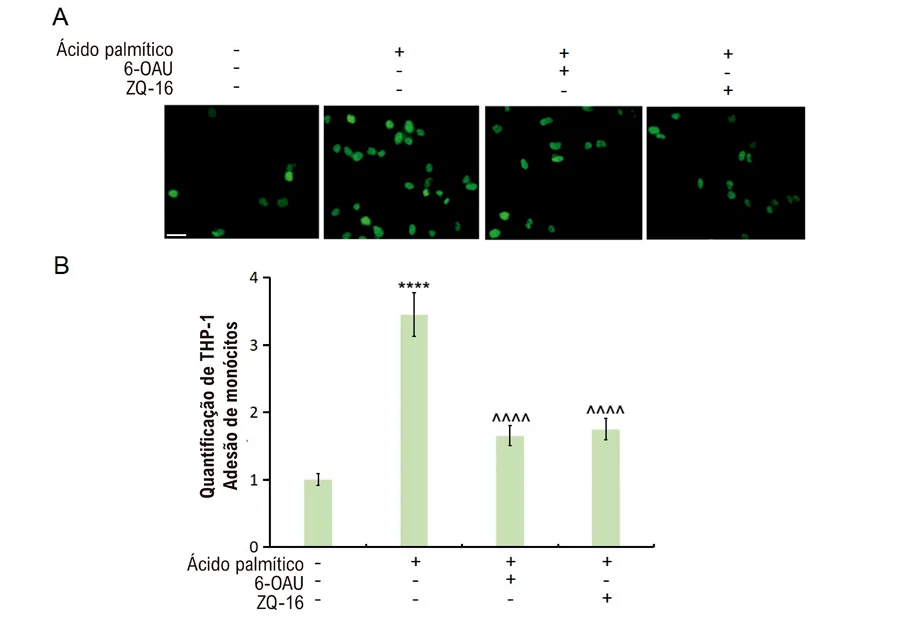
– A ativação do GPR84 inibe a adesão de monócitos a monocamadas endoteliais. Células endoteliais da aorta humana (HAECs) pré-tratadas por 24 horas com 1 mM de PA ± agonistas de GPR84 foram cocultivadas por 1 hora com monócitos THP-1 carregados com éster acetoximetil de calceína (calceína-AM). (A) Micrografias de fluorescência representativas mostrando células aderentes (barra de escala, 50 μm). (B) Quantificação de monócitos ligados, normalizada aos controles não tratados. ****p < 0,0001 versus controles não tratados; ^^^^p < 0,0001 versus o grupo tratado apenas com PA (ANOVA de uma via com teste post-hoc de Tukey).

### Supressão das vias de sinalização NF-κB

Investigamos então os mecanismos de sinalização intracelular que regem esses efeitos anti-inflamatórios, com foco específico na via de transcrição do fator nuclear κB (NF-κB), dada a sua função estabelecida na regulação da expressão de genes inflamatórios. A análise por Western blot de extratos de proteínas nucleares demonstrou que o tratamento com ácido palmítico promoveu uma translocação nuclear significativa da subunidade p65 do NF-κB, e esse efeito foi substancialmente atenuado em células que receberam tratamento concomitante com o agonista de GPR84 (Figura 5A). Ensaios com repórter de luciferase utilizando construções de promotores específicos para NF-κB confirmaram ainda que o tratamento com o agonista suprimiu acentuadamente a atividade transcricional do NF-κB em comparação com a estimulação apenas com ácido palmítico (Figura 5B). Essas observações mecanísticas sugerem que a sinalização de GPR84 interfere nas vias canônicas de ativação do NF-κB, atenuando, assim, a transcrição de genes inflamatórios a jusante.

**Figura 5:**
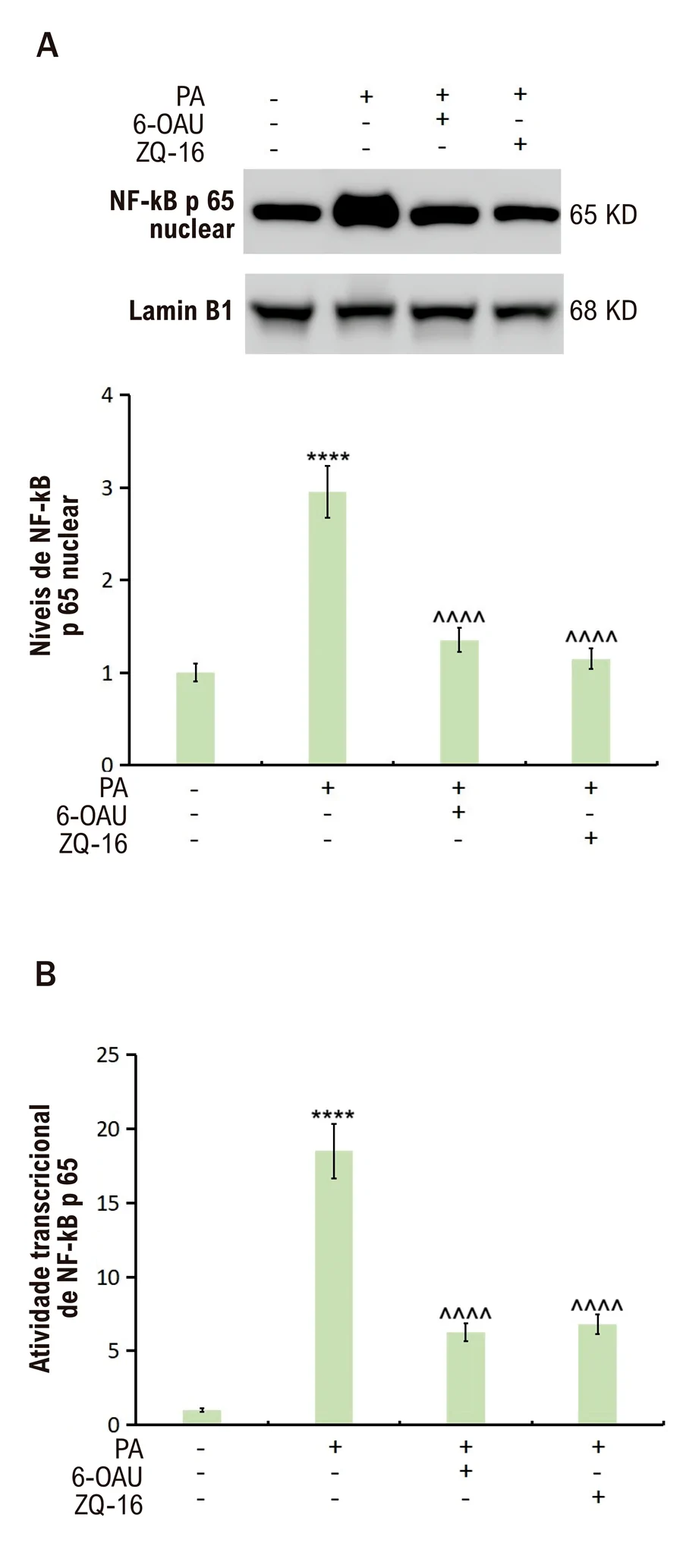
– A ativação do GPR84 suprime a ativação do NF-κB (fator nuclear kappa B). Células endoteliais da aorta humana (HAECs) foram estimuladas por 24 horas com 1 mM de ácido palmítico (PA), isoladamente ou em conjunto com 1 μM de 6-n-octilaminouracila (6-OAU) ou 1 μM de ZQ-16. (A) Frações nucleares foram submetidas a immunoblotting para p65; Lamin B1 serviu como controle de carregamento. (B) Atividade da luciferase induzida por NF-κB normalizada ao controle com Renilla. ****p < 0,0001 em comparação com os controles não tratados; ^^^^p < 0,0001 versus o grupo tratado apenas com PA (ANOVA de uma via com teste post-hoc de Tukey).

### Preservação da expressão de Gfi1 pela ativação de GPR84

Nossa hipótese era de que o fator de crescimento independente 1 (GFI1), um repressor transcricional, poderia participar da citoproteção mediada por GPR84 por meio de interações previamente caracterizadas com componentes da sinalização de NF-κB. A exposição ao ácido palmítico diminuiu significativamente a abundância de mRNA de Gfi1 em comparação com os controles não tratados, enquanto o tratamento concomitante com 6-OAU ou ZQ-16 manteve os níveis de expressão comparáveis aos basais (Figura 6A). A análise por immunoblot utilizando anticorpos específicos para Gfi1 validou que os níveis de proteína seguiram padrões de expressão semelhantes, com o pré-tratamento com o agonista prevenindo efetivamente a depleção de Gfi1 induzida pelo ácido palmítico (Figura 6B). Essas observações corroboram a premissa de que a ativação de GPR84 preserva a expressão de Gfi1 em condições inflamatórias, potencialmente mantendo a repressão transcricional de genes-alvo pró-inflamatórios.

**Figura 6:**
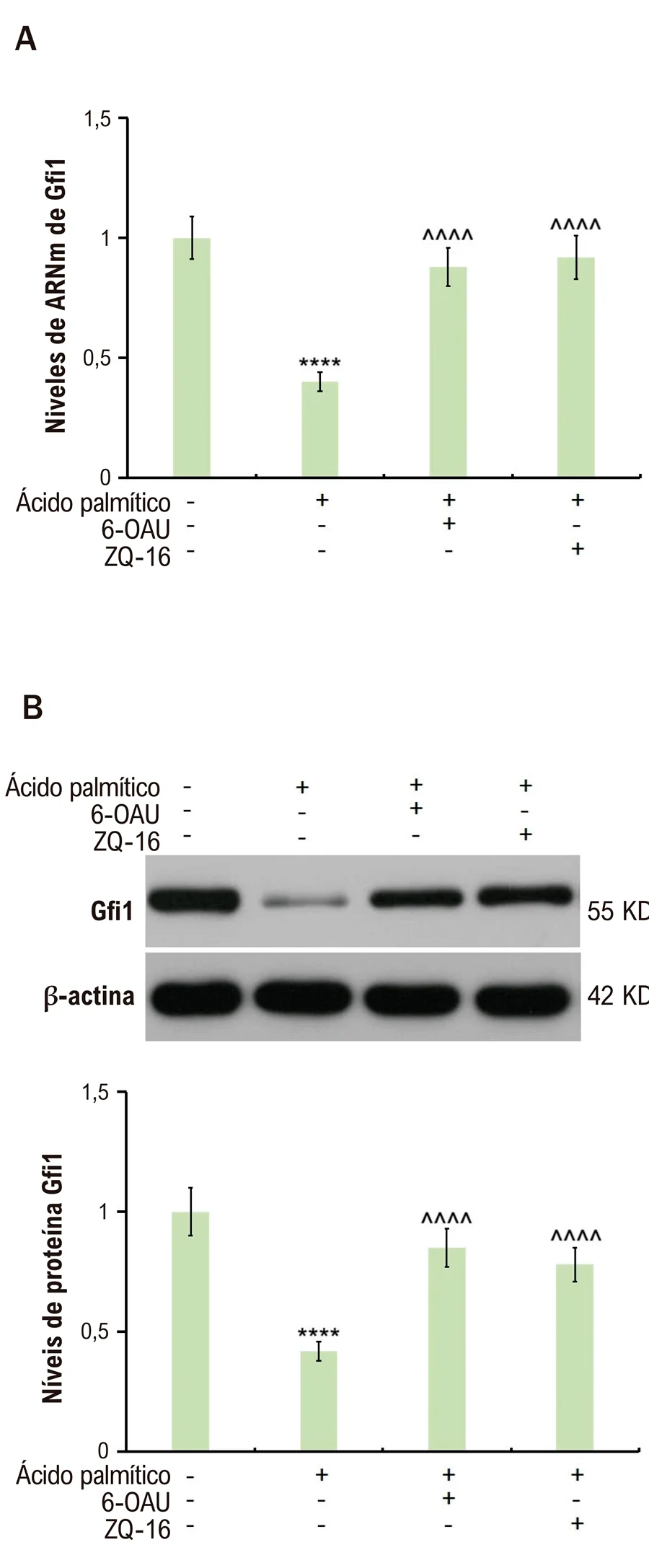
– A sinalização de GPR84 mantém a expressão do fator de crescimento independente 1 (Gfi1) em condições inflamatórias. Células endoteliais da aorta humana (HAECs) foram incubadas por 24 horas com 1 mM de PA ± agonistas de GPR84. (A) mRNA de Gfi1 quantificado por qPCR e normalizado para 18S rRNA. (B) Imunoblotagens representativas e análise densitométrica da proteína Gfi1 em relação à β-actina. ****p < 0,0001 em comparação com os controles não tratados; ^^^^p < 0,0001 versus o grupo tratado apenas com PA (ANOVA de uma via com teste post-hoc de Tukey).

### Necessidade de Gfi1 para efeitos protetores mediados por GPR84

Para estabelecer definitivamente se Gfi1 é necessário para a eficácia do agonista, em vez de estar meramente associado a respostas protetoras, empregamos interferência de RNA para deplecionar Gfi1 antes da estimulação farmacológica com agonistas do receptor. A entrega mediada por adenovírus de RNA em grampo curto (*short hairpin RNA*) específico para Gfi1 resultou em *knockdown* proteico eficiente, reduzindo a imunorreatividade da Gfi1 em aproximadamente 70% em comparação com sequências controle aleatórias (Figura 7A).

**Figura 7:**
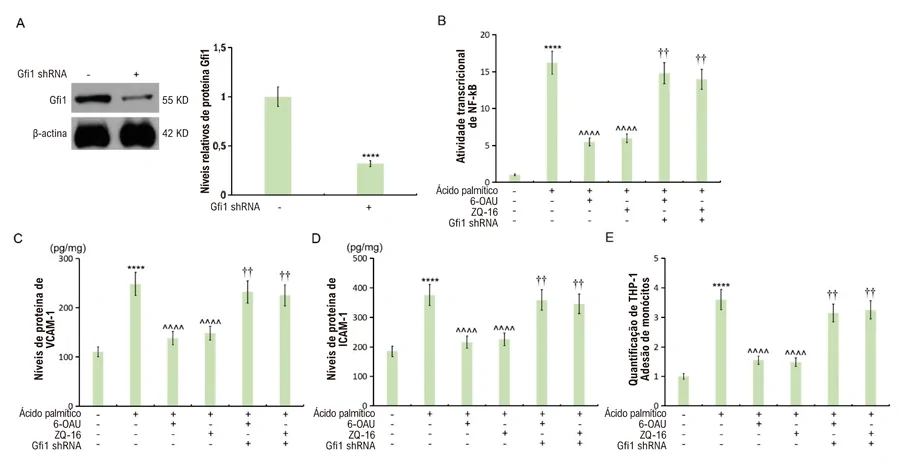
– A Depleção de Gfi1 reverte a proteção mediada por agonistas. Células endoteliais da aorta humana (HAECs) foram transduzidas por 48 horas com adenovírus codificando RNA de interferência curto (shRNA) direcionado a Gfi1 ou shRNA controle não específico, e então estimuladas por 24 horas com 1 mM de ácido palmítico (PA) ± 1 μM de agonistas de GPR84 (6-OAU ou ZQ-16). (A) Imunoblot representativo e quantificação confirmando o knockdown eficiente de Gfi1 (****p < 0,0001 comparado ao grupo shRNA controle não específico). (B) Atividade do repórter de luciferase do fator nuclear κB (NF-κB) normalizada ao controle de Renilla. (C, D) Níveis de proteína da molécula de adesão celular vascular-1 (VCAM-1) e da molécula de adesão intercelular-1 (ICAM-1) secretadas, medidos por ensaio imunoenzimático (ELISA). (E) Quantificação da adesão de monócitos THP-1 marcados com calceína-AM. Para os painéis BE: ****p < 0,0001 em comparação com o grupo controle não tratado; ^^^^p < 0,0001 em comparação com o grupo tratado apenas com PA; ††p < 0,01 em comparação com o grupo PA + agonista correspondente (ANOVA de duas vias com teste post-hoc de Šidák).

Nessas condições de silenciamento, a capacidade de 6-OAU e ZQ-16 de suprimir a atividade transcricional de NF-κB foi abolida (Figura 7B). De forma semelhante, a depleção de Gfi1 reverteu as reduções mediadas por agonistas na secreção da proteína VCAM-1 (Figura 7C) e na produção da proteína ICAM-1 (Figura 7D). Ensaios funcionais de adesão confirmaram posteriormente que, sem a expressão intacta de Gfi1, os agonistas de GPR84 não conseguiram atenuar a adesão de monócitos a monocamadas endoteliais (Figura 7E). Em conjunto, esses experimentos de resgate confirmam que Gfi1 é indispensável para os efeitos anti-inflamatórios e antiadesivos mediados por GPR84, estabelecendo esse fator de transcrição como um mediador proximal crítico na cascata de sinalização.

## Discussão

A presente investigação descreve uma cascata de sinalização citoprotetora na qual a ativação do GPR84 atenua a inflamação endotelial induzida pelo ácido palmítico através da preservação da expressão de Gfi1 e subsequente inibição da atividade transcricional do fator nuclear-κB.^23^ Estas observações divergem substancialmente de caracterizações anteriores que descreviam o GPR84 como um receptor predominantemente pró-inflamatório em linhagens mieloides, estabelecendo assim uma diversidade funcional dependente do contexto que exige uma análise mecanística cuidadosa.^18^

A Figura central é uma ilustração esquemática do mecanismo protetor do agonismo do GPR84 contra a inflamação endotelial induzida por AGLs. A ativação do GPR84 preserva a expressão de Gfi1, que, por sua vez, inibe a translocação nuclear de NF-κB p65, levando à redução da expressão de citocinas pró-inflamatórias (IL-1β, TNF-α, MCP-1) e moléculas de adesão (VCAM-1, ICAM-1), atenuando, em última análise, a adesão de monócitos ao endotélio.

**Figura Central:**
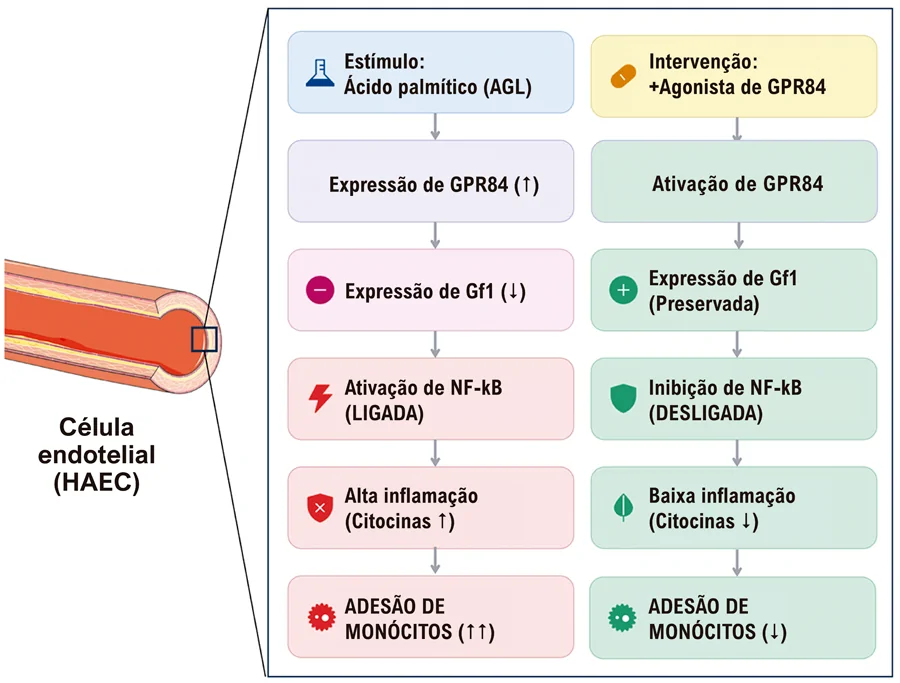
: O Agonismo do GPR84 Atenua a Inflamação Endotelial Induzida por Ácidos Graxos Livres e a Adesão de Monócitos através da Via Gfi1/NF-Κb

Investigações anteriores demonstraram que a Nesfatina-1 protege as HAECs contra a inflamação induzida por AGLs através da supressão idêntica da sinalização do fator nuclear kappa B dependente de Gfi1.^23^ O presente estudo amplia esse paradigma mecanístico ao identificar o GPR84 como um gatilho farmacológico a montante capaz de ativar essa via protetora. Embora ambas as investigações utilizem modelos celulares e estímulos inflamatórios idênticos, o presente trabalho identifica, de forma inédita, um alvo receptor passível de ser alvo de fármacos, que responde a agonistas sintéticos com propriedades farmacêuticas favoráveis.^23^

Os efeitos anti-inflamatórios do agonismo do GPR84 observados em células endoteliais vão além das funções pró-inflamatórias canônicas previamente atribuídas a esse receptor em células mieloides, ressaltando a importância do contexto celular na determinação dos resultados da sinalização do GPR8424. Recio et al. demonstraram que o agonismo do GPR84 aumenta a secreção de citocinas, a quimiotaxia e a atividade fagocítica em macrófagos sob condições inflamatórias, sugerindo que o receptor amplifica as respostas imunes inatas.^24^ De forma semelhante, Nicol et al. demonstraram que o GPR84 medeia a hipersensibilidade mecânica e a hipersensibilidade térmica em modelos de dor neuropática através da ativação de macrófagos periféricos.^17^ Essas ações pró-inflamatórias motivaram o desenvolvimento de antagonistas do GPR84 para colite ulcerativa, doenças fibróticas e dor neuropática.^25^ No entanto, investigações recentes revelam que a função do GPR84 varia significativamente dependendo do contexto celular e do ambiente metabólico.^26^

Os resultados atuais estão em consonância com evidências emergentes de que o GPR84 funciona como um receptor imunomodulador, em vez de um simples mediador pró-inflamatório.^26^ Em condições lipotóxicas caracterizadas por exposição excessiva a ácidos graxos saturados, a ativação do GPR84 parece suprimir, em vez de amplificar, a sinalização inflamatória, representando potencialmente um mecanismo homeostático compensatório.^23^ Essa funcionalidade dependente do contexto pode explicar as aparentes contradições entre os presentes achados endoteliais e estudos anteriores com macrófagos, uma vez que o ambiente metabólico e o estado de diferenciação celular provavelmente determinam os resultados da sinalização subsequente.^26^

A identificação de Gfi1 como um mediador crítico a jusante da citoproteção mediada por GPR84 constitui um avanço mecanístico significativo.^27^ Investigações anteriores estabeleceram Gfi1 como um repressor transcricional com domínio de dedo de zinco que antagoniza a ligação do fator nuclear kappa B p65 ao DNA, limitando assim a produção de citocinas inflamatórias.^27^ No entanto, esses estudos anteriores focaram-se predominantemente em linhagens hematopoiéticas, deixando o papel funcional de Gfi1 em células endoteliais sob estresse metabólico em grande parte inexplorado.^28^ A presente demonstração de que a redução da expressão de Gfi1 reverte a proteção mediada por GPR84 confirma que esse fator de transcrição opera como um nó de sinalização não redundante no endotélio vascular.^27^

Diversas limitações restringem a interpretação destes resultados. A investigação baseou-se exclusivamente em modelos de cultura celular in vitro utilizando monocamadas bidimensionais estáticas, que não apresentam o estresse de cisalhamento hemodinâmico fisiológico, a complexidade multicelular e a arquitetura tridimensional características da vasculatura arterial.^28^ Os modelos convencionais de cultura de tecidos em plástico demonstram baixa relevância fisiológica para a pesquisa em aterosclerose, uma vez que não conseguem replicar os estágios iniciais da formação da placa, a dinâmica da permeabilidade endotelial ou as interações com os componentes sanguíneos circulantes.^29^ Investigações futuras devem empregar tecnologias microfluídicas de órgãos em chip que mimetizem o fluxo de cisalhamento vascular e permitam o cocultivo com células musculares lisas e monócitos em condições dinâmicas.^29^ Além disso, nossos experimentos foram restritos a HAECs. A verificação do eixo GPR84–Gfi1–NF-κB em outros subtipos endoteliais, como células endoteliais da veia umbilical humana (HUVECs) ou HAECs, será necessária para estabelecer uma relevância vascular mais ampla.

Além disso, o estudo utilizou concentrações suprafisiológicas de ácido palmítico que podem induzir citotoxicidade não específica em vez de ativação inflamatória seletiva, limitando potencialmente a relevância translacional.^25^ A ausência de validação in vivo representa outra limitação crítica, visto que a aterogênese envolve interações multicelulares complexas entre o endotélio, leucócitos circulantes e células musculares lisas vasculares, que não podem ser reproduzidas em sistemas de monocultura.^25^ Estudos em animais utilizando modelos alimentados com dieta rica em gordura e com manipulação específica do GPR84 no endotélio forneceriam confirmação essencial do mecanismo protetor observado in vitro.^17^ Ademais, apenas dois agonistas sintéticos do GPR84 (6-OAU e ZQ-16) foram examinados; a validação com um painel mais amplo de agonistas estruturalmente distintos reforçaria a generalização dos efeitos protetores e ajudaria a descartar potenciais atividades fora do alvo.

Investigações futuras devem explorar a ligação molecular precisa entre a ativação do receptor GPR84 e a regulação transcricional positiva de Gfi1.^30^ Os mecanismos potenciais podem envolver a inibição da adenilato ciclase mediada por Gi/o, a modulação subsequente da atividade da proteína quinase A e alterações nos estados de fosforilação do fator de transcrição que aumentam a acessibilidade do promotor de Gfi1.^30^ Além disso, o potencial terapêutico dos agonistas de GPR84 para a proteção vascular em distúrbios metabólicos requer avaliação em modelos pré-clínicos de aterosclerose, com atenção especial aos potenciais efeitos divergentes nas populações de células endoteliais versus mieloides.^23^ O desenvolvimento de sistemas de administração específicos para tecidos ou agonistas direcionados que ativem seletivamente as vias de proteção endotelial, minimizando as respostas pró-inflamatórias dos macrófagos, pode otimizar os índices terapêuticos.^26^

## Conclusão

Em resumo, esses achados estabelecem o GPR84 como um modulador da inflamação endotelial dependente do contexto, que opera por meio de um mecanismo dependente de Gfi1, distinto de suas funções caracterizadas em células imunes. Esta investigação amplia a compreensão da detecção metabólica no endotélio vascular e identifica um novo eixo receptor-fator de transcrição com potenciais implicações terapêuticas para doenças vasculares ateroscleróticas.
