# Supplementary material for: O Agonismo do GPR84 Atenua a Inflamação Endotelial Induzida por Ácidos Graxos Livres e a Adesão de Monócitos através da Via Gfi1/NF-Κb
Source: Arq Bras Cardiol. 2026 Sep 10;123(8):e20260231. [Article in Portuguese] doi: 10.36660/abc.20260231 (PMC13671130; doi:10.36660/abc.20260231)
Supplement: Translation

# Translation: Agonism of GPR84 Attenuates Free Fatty Acid-Induced Endothelial Inflammation and Monocyte Adhesion via Gfi1/NF-κB Pathway

**DOI:** 10.36660/abc.20260231i

**Authors:** Li Li, Gang Fu, Yanling Liu, Caiyun Liu

**Affiliations:** 1 China Department of Cardiology, The Third People’s Hospital of Longgang District Shenzhen, Guangdong Province – China

**Keywords:** Atherosclerosis, Vascular Endothelium, Monocytes

## Abstract

**Background:**

Endothelial dysfunction and monocyte adhesion driven by free fatty acids (FFAs) are critical early events in atherosclerosis. Palmitate, a long-chain saturated free fatty acid, upregulated GPR84 expression. Although GPR84 is canonically activated by medium-chain FFAs, its induction under lipotoxic stress suggests a compensatory, context-dependent role. Its role in endothelial inflammation is poorly defined.

**Objectives:**

To investigate the functional role of GPR84 activation in FFA-induced endothelial inflammation and elucidate the underlying mechanisms.

**Methods:**

Human aortic endothelial cells (HAECs) were challenged with palmitate (0.5 or 1 mM) in the presence or absence of the GPR84 agonists 6-OAU or ZQ-16 (each at 1 μM). Inflammatory cytokine and adhesion molecule expression were assessed by qPCR and ELISA. Monocyte adhesion was evaluated using THP-1 cells. NF-κB p65 nuclear translocation and Gfi1 expression were examined, and Gfi1 silencing was performed to assess functional relevance.

**Results:**

Palmitate increased GPR84 expression in HAECs. Treatment with GPR84 agonists significantly reduced palmitate-induced upregulation of IL-1β, TNF-α, MCP-1, VCAM-1, and ICAM-1 at both mRNA and protein levels, and attenuated THP-1 monocyte adhesion. Mechanistically, GPR84 activation inhibited NF-κB p65 nuclear translocation and preserved Gfi1 expression. Knockdown of Gfi1 abrogated the protective effects of GPR84 agonists, restoring NF-κB activation, adhesion molecule expression, and monocyte adhesion.

**Conclusions:**

GPR84 agonism mitigates FFA-induced endothelial inflammation by restoring Gfi1 expression and suppressing NF-κB signaling, highlighting a potential therapeutic strategy for early atherosclerosis that warrants further in vivo validation.

## Introduction

Cardiovascular pathologies underlying myocardial infarction, stroke, and peripheral artery disease collectively represent the predominant mortality source across developed and developing nations.^1^ Arterial walls are lined by a single layer of metabolically active endothelial cells that coordinate vasomotor function, leukocyte adhesion, and inflammatory cascades to preserve vascular homeostasis.^2^ Metabolic changes associated with obesity and diabetes mellitus cause endothelial activation, converting this protective barrier into a pro-inflammatory surface that attracts circulating leukocytes.^3^ Oxidative stressors and modified lipoproteins, including oxidized low-density lipoprotein (ox-LDL), accumulate in the subendothelial intima, causing phenotypic changes and increasing the production of sticky glycoproteins that promote monocyte anchoring and subsequent transmigration.^4^

Free fatty acids (FFAs) at increased quantities under dyslipidemic conditions directly damage endothelial integrity by generating reactive oxygen species and activating pattern recognition receptors.^5^ Palmitic acid, a sixteen-carbon saturated fatty acid included in dietary lipids, stimulates endothelial activation as seen by increased production of vascular cell adhesion molecule-1 and intercellular adhesion molecule-1.^6^ In human aortic endothelial cells (HAECs), palmitate exposure triggers NF-κB nuclear translocation and transcriptional upregulation of multiple pro-inflammatory mediators, including IL-1β, TNF-α, and MCP-1.^7^ These molecular changes improve endothelial adhesive capabilities, which promote circulating monocyte adhesion and diapedesis into the artery wall.^8^

Monocyte adherence to active endothelium surfaces is a major rate-limiting step in atheroma formation.^9^ Following strong attachment, monocytes cross the endothelial barrier and develop into macrophages in the intimal layer, where they ingest oxidized lipoproteins and convert into cholesterol-rich foam cells.^10^ The nuclear factor-kappa B transcription factor family coordinates endothelial activation by regulating the production of adhesion molecules and soluble inflammatory mediators in response to cytokines or lipid-derived molecular patterns.^11^ Canonical nuclear factor-kappa B signaling includes phosphorylation of inhibitory proteins, nuclear translocation of p65-p50 heterodimers, and promoter binding at kappa B consensus sites found in many pro-inflammatory genes.^12^ Homeostatic negative feedback mechanisms ordinarily stop inflammatory reactions, but prolonged metabolic stress seems to disrupt these regulatory systems, leading to persistent endothelial activation and progressive vascular damage.^13^

G-protein-coupled receptor 84 has been identified as a possible regulator of inflammatory responses via selective activation by medium-chain FFAs.^14^ GPR84 was first identified as an orphan receptor expressed only in myeloid lineages, but subsequent structural investigations show that it links to heterotrimeric Gi/o proteins, lowering intracellular cyclic adenosine monophosphate concentrations upon agonist engagement.^15^ Pharmacological studies show that synthetic agonists such as 6-OAU and ZQ-16 influence macrophage inflammatory polarization and neutrophil effector activities.^16^ Studies using genetic deletion models show that this receptor has a role in wound healing, mucosal inflammation, and neuropathic pain, although its function inside the vascular endothelium is still poorly understood.^17^ The expression patterns and functional ramifications of GPR84 activation in HAECs treated with saturated fatty acids have not been thoroughly investigated. Although palmitate falls outside the typical medium-chain binding range of GPR84, GPR84 is known to modulate cellular responses to lipotoxic stress induced by long-chain saturated fatty acids.^18^

Growth factor independence 1 (Gfi1) is a transcriptional repressor with zinc fingers that regulates innate immune responses and hematopoietic differentiation.^19^ Previous research has shown that Gfi1 reduces nuclear factor-κB activity by blocking p65 DNA binding at target gene promoters, reducing inflammatory cytokine production.^20^ Toll-like receptor activation induces Gfi1 expression, which forms part of a negative feedback loop that limits excessive inflammatory reactions.^21^ Recent studies show that Gfi1 loss in macrophages causes spontaneous inflammatory activation characterized by increased nuclear factor-κB signaling and cytokine release.^22^ While Gfi1 has been extensively researched in hematological lineages, its expression and functional involvement in endothelial cells subjected to metabolic stress are less understood.

We anticipated that GPR84 activation influences palmitic acid-induced endothelial inflammation by regulating Gfi1 expression and inhibiting nuclear factor-κB signaling. This study shows that palmitic acid stimulates GPR84 expression in HAECs; however, pharmaceutical GPR84 agonism reduces inflammatory cytokine production, adhesion molecule expression, and monocyte adherence. Mechanistic investigations show that activating GPR84 retains Gfi1 expression in inflammatory circumstances, but genetic deletion of Gfi1 reverses the protective benefits of receptor agonism. These results establish a new GPR84-Gfi1-nuclear factor-κB axis that modulates endothelial inflammatory responses and might be a therapeutic target for vascular protection in metabolic diseases.

## Materials and methods

### Reagents and antibodies

Palmitic acid (free acid, P0500), fatty acid-free bovine serum albumin (A8806), and all general laboratory chemicals were procured from Sigma-Aldrich unless otherwise specified. Synthetic GPR84 agonist 6-OAU (13583) was obtained from Cayman Chemical, and GPR84 agonist ZQ-16 (HY-112042) was obtained from MedChemExpress. The following primary antibodies were employed for immunodetection: mouse monoclonal anti-GPR84 (Santa Cruz Biotechnology, sc-514951), rabbit monoclonal antibodies against NF-κB p65 (Cell Signaling Technology, 8242), total Gfi1 (Cell Signaling Technology, 31929), and Lamin B1 (Cell Signaling Technology, 13435), together with rabbit polyclonal antibody against β-actin (Cell Signaling Technology, 4967). Secondary antibodies conjugated to horseradish peroxidase included anti-rabbit IgG (Cell Signaling Technology, 7074) and anti-mouse IgG (Cell Signaling Technology, 7076). Commercial ELISA development systems for quantifying human IL-1β (DY201), TNF-α (DY210), MCP-1 (DY279), VCAM-1 (DY809), and ICAM-1 (DY720) were acquired from R&D Systems. Adenoviral vectors encoding Gfi1-targeting shRNA (sc-35467-V) and scrambled control shRNA (sc-108080) were supplied by Santa Cruz Biotechnology.

### Cell culture and maintenance

Primary HAECs (Lonza, CC-2535) were cultured in EGM-2 medium (Lonza, CC-3162) supplemented with the manufacturer’s provided growth factors, including 2% FBS, hydrocortisone, hFGF, VEGF, R3-IGF-1, ascorbic acid, hEGF, and GA-1000. Incubation conditions were maintained at 37°C with 5% CO_2_ in a humidified chamber, and cells were used up to passage six. THP-1 monocytes (ATCC, TIB-202) were maintained in RPMI 1640 (Gibco, 11875093) containing 10% FBS, 2 mM L-glutamine, 100 U/mL penicillin, and 100 μg/mL streptomycin. To avoid spontaneous differentiation, cell densities were kept within 2×10^5^ to 1×10^6^ cells/mL throughout culture. All cell lines were tested for mycoplasma contamination using the MycoAlert Mycoplasma Detection Kit (Lonza, LT07-218) at passage initiation and every four weeks thereafter; all cultures used in this study were confirmed negative.

### Preparation of fatty acid-albumin complexes

To prepare palmitic acid for cell culture experiments, we first dissolved the free acid in 0.1 M NaOH (filter-sterilized) at 70°C under constant stirring until the solution clarified. After cooling, the pH was titrated to 7.4 using 1 M HCl, and the resulting stock was passed through a 0.22 μm filter to ensure sterility. For experimental use, this 100 mM stock was complexed with fatty acid-free BSA in serum-free basal medium at a 5:1 molar ratio, yielding final working concentrations of 0.5 mM or 1 mM palmitic acid immediately before cell treatment. The final BSA concentration was approximately 0.834% (w/v) at the 1 mM palmitate working concentration, corresponding to the 5:1 molar ratio. Complexes were utilized immediately upon preparation to prevent precipitation. Before applying the palmitate-BSA complexes, cells were maintained for 12 hours in medium containing 2% FBS to reduce baseline activation, as described in the Pharmacological Stimulation section.

### Pharmacological stimulation and shRNA transduction

Before experimental treatments, confluent HAEC monolayers were serum-starved for 12 hours in EGM-2 medium containing 2% FBS to minimize basal activation. Cells were then exposed to palmitic acid-BSA conjugates (0.5 mM or 1 mM final) for 24 hours, either alone or concurrently with the GPR84 agonists 6-OAU (1 μM) or ZQ-16 (1 μM). For loss-of-function studies, HAECs were first incubated for 48 hours with adenoviruses encoding Gfi1-targeting shRNA or scrambled control sequences at an MOI of 50. Knockdown efficiency was verified by Western blot analysis, which showed an approximately 70% reduction in Gfi1 protein levels.

### RNA extraction and quantitative Real-Time PCR

We extracted total RNA from cultured cells using TRIzol Reagent (Invitrogen, 15596026) following the manufacturer’s supplied protocol. First-strand cDNA was reverse-transcribed from 1 μg of purified RNA using the iScript cDNA Synthesis Kit (Bio-Rad, 1708891). Quantitative PCR reactions were performed with SYBR Green PCR Master Mix (Applied Biosystems, 4368577) on a QuantStudio 3 instrument (Applied Biosystems). Thermocycling conditions were: 95°C for 10 min, followed by 40 cycles of 95°C for 15 s and 60°C for 1 min, with a final melt curve step. Primer sequences for the following human genes were obtained from the PrimerBank database: GPR84 (F: 5’-TTGGCATCTTCTATTGCCTCATC-3’, R: 5’-TGTCGCAACTTGTATTGGTCC-3’); IL-1β (F: 5’-AAACAGATGAAGTGCTCCTTCCAGG-3’, R: 5’-TGGAGAACACCACTTGTTGCTCCA-3’); TNF-α (F: 5’-GAGGCCAAGCCCTGGTATG-3’, R: 5’-CGGGCCGATTGATCTCAGC-3’); MCP-1 (F: 5’-CAGCCAGATGCAATCAATGCC-3’, R: 5’-TGGAATCCTGAACCCACTTCT-3’); VCAM-1 (F: 5’-GGGAAGATGGTCGTGATCCTT-3’, R: 5’-TCTGGGGTGGTCTCGATTTTA-3’); ICAM-1 (F: 5’-ATGCCCAGACATCTGTGTCC-3’, R: 5’-GGGGTCTCTATGCCCAACAA-3’); Gfi1 (F: 5’-CCGCGCTCATTTCTCGTCA-3’, R: 5’-ACGGAGGGAATAGTCTGGTCC-3’). 18S rRNA (F: 5’-GTAACCCGTTGAACCCCATT-3’, R: 5’-CCATCCAATCGGTAGTAGCG-3’) was used as the internal reference for normalization. Target gene expression relative to the 18S rRNA control was calculated using the comparative Ct method (2^-^).

### Protein extraction and immunoblot analysis

We prepared whole-cell lysates by harvesting cells in RIPA buffer (Thermo, 89900) containing protease and phosphatase inhibitor cocktail (Thermo, A32959). Nuclear and cytoplasmic fractions were separated using the NE-PER extraction system (Thermo, 78833) according to the instructions provided by the manufacturer. Protein concentrations in each sample were measured using the BCA assay (Pierce, 23225). Protein samples (20 μg per lane) were resolved by SDS-PAGE on 10% polyacrylamide gels and then electrotransferred to PVDF membranes (Millipore, IPVH00010). To minimize non-specific binding, membranes were incubated for 1 hour at room temperature in blocking buffer (5% non-fat milk in TBST), followed by overnight incubation at 4°C with primary antibodies diluted 1:1000 in blocking solution. After extensive washing, the membranes were incubated for 1 hour at room temperature with HRP-conjugated secondary antibodies (1:2000 dilution). β-actin was used as the loading control for whole-cell lysates, whereas Lamin B1 served as the loading control for nuclear fractions. Protein bands were detected using Pierce ECL substrate (Thermo, 32106), and signal intensities were quantified by densitometry with ImageJ software (NIH).

### Cytokine and adhesion molecule quantification

We measured secreted protein concentrations for IL-1β, TNF-α, MCP-1, VCAM-1, and ICAM-1 in collected culture supernatants using DuoSet ELISA kits (R&D Systems) according to the manufacturer’s recommended procedures. Absorbance readings were taken at 450 nm with background correction at 540 nm on a BioTek Synergy H1 microplate reader. Sample concentrations were interpolated from standard curves and then normalized to total protein content in corresponding cell lysates, expressed as picograms per milligram of protein.

### Monocyte adhesion assay

THP-1 cells were harvested, washed, and resuspended in serum-free RPMI 1640 at a density of 2 × 10^5^ cells per milliliter. Cell suspensions were incubated with 1 μM calcein-AM (Invitrogen C3099) for 30 minutes at 37°C with intermittent mixing, followed by three washes with phosphate-buffered saline to remove unincorporated dye. Labeled THP-1 cells (2 × 10^5^ cells per well) were co-incubated with treated HAEC monolayers within twenty-four-well plates for exactly one hour at 37°C. Non-adherent cells were removed by gentle aspiration of medium followed by two rapid washes with 1 mL pre-warmed (37°C) phosphate-buffered saline per well. Adherent cells were visualized immediately using an inverted fluorescence microscope (excitation 494 nm, emission 517 nm) equipped with a 10× objective. Representative images were captured for each experimental condition. For quantitative analysis, attached cells were counted in five random fields per well utilizing ImageJ software (National Institutes of Health) by an investigator blinded to treatment groups, and results were expressed as relative fold change compared with untreated controls.

### Luciferase reporter gene assay

HAECs were plated at a density of 1 × 10^5^ cells per well in twenty-four-well plates twenty-four hours before transfection. Cells underwent transient transfection with NF-κB firefly luciferase reporter plasmid (pNF-κB-Luc, Promega, Cat.# E8491) and Renilla luciferase control vector (pRL-TK, Promega E2241) using Lipofectamine 3000 (Invitrogen L3000015) per supplier specifications. Following twenty-four hours of recovery, transfected cells underwent serum starvation and subsequent pharmacological treatment as described. Cellular lysates were prepared utilizing Passive Lysis Buffer (Promega E1941). Firefly and Renilla luciferase activities were measured sequentially utilizing the Dual-Luciferase Reporter Assay System (Promega E1910) on a luminometer (Turner BioSystems Modulus). NF-κB transcriptional activity was calculated as the ratio of firefly to Renilla luciferase activity and normalized to the untreated control.

### Statistical analysis

All quantitative data are expressed as the mean ± standard error of the mean (SEM) derived from at least three independent experiments performed in duplicate or triplicate. Before ANOVA, data were assessed for normality using the Shapiro-Wilk test and for homogeneity of variances using Levene’s test. All datasets satisfied the assumptions required for parametric analysis. To determine statistical significance, we applied one-way ANOVA followed by appropriate post-hoc tests (Tukey’s, Dunnett’s, or Holm-Šidák) for multiple group comparisons, or two-way ANOVA with Šidák’s correction for experiments incorporating two independent variables. Differences were considered statistically significant at P < 0.05. All statistical calculations and graph generation were carried out using GraphPad Prism version 9.0 (GraphPad Software, La Jolla, CA).

## Results

### GPR84 induction by palmitic acid in human aortic endothelial cells after 24-hour exposure

We first sought to determine whether palmitic acid, a representative pro-inflammatory long-chain saturated fatty acid, modulates the expression of GPR84 after 24-hour exposure in HAECs under controlled experimental conditions. Exposure to palmitic acid at concentrations of 0.5 mM and 1 mM for twenty-four hours produced concentration-dependent increases in GPR84 transcript abundance relative to untreated controls, as quantified by real-time polymerase chain reaction (Fig. 1A). Concurrent Western blot analyses corroborated these transcriptional alterations at the protein level, demonstrating marked upregulation of GPR84 immunoreactivity following identical stimulation protocols (Fig. 1B). These data indicate that palmitic acid induces GPR84 expression through mechanisms operating at both transcriptional and protein levels.

**Figure 1:** – Palmitic acid induces GPR84 expression in human aortic endothelial cells (HAECs). Cells were treated with 0.5 or 1 mM palmitic acid for 24 hours. (A) Quantitative PCR (qPCR) showing GPR84 mRNA levels normalized to 18S rRNA. (B) Representative immunoblots and quantification of GPR84 protein levels relative to β-actin. **p < 0.01, ****p < 0.0001 compared with untreated controls; #p < 0.05 between 0.5 mM and 1 mM palmitic acid groups (one-way ANOVA with Tukey’s post-hoc test).

### Attenuation of pro-inflammatory cytokine production via GPR84 agonism

We next examined whether pharmacological activation of GPR84 using structurally distinct synthetic agonists attenuates the pro-inflammatory cytokine cascade initiated by palmitic acid challenge. Quantitative real-time PCR analyses revealed that pretreatment with either 6-OAU or ZQ-16 substantially diminished palmitic acid-induced upregulation of interleukin-1β mRNA (Figure 2A). Analogous inhibitory patterns emerged for tumor necrosis factor-α transcript levels (Fig. 2B) and monocyte chemoattractant protein-1 mRNA expression (Figure 2C). Enzyme-linked immunosorbent assays performed on conditioned culture media confirmed that these transcriptional modifications translated into corresponding reductions in secreted IL-1β protein concentrations (Figure 2D), TNF-α protein levels (Figure 2E), and MCP-1 protein abundance (Figure 2F). Collectively, these findings demonstrate that GPR84 agonism suppresses palmitic acid-driven inflammatory mediator production through coordinated regulation of gene expression and protein secretion.

**Figure 2:** – GPR84 agonists attenuate pro-inflammatory cytokine production. Human aortic endothelial cells (HAECs) were stimulated for 24 hours with 1 mM palmitic acid (PA), either alone or together with 1 μM 6-n-octylaminouracil (6-OAU) or 1 μM ZQ-16. (A–C) Relative mRNA expression of interleukin-1β (IL-1β), tumor necrosis factor-α (TNF-α), and monocyte chemoattractant protein-1 (MCP-1) measured by qPCR. (D–F) Corresponding secreted protein concentrations in culture supernatants were quantified by enzyme-linked immunosorbent assay (ELISA). ****p < 0.0001 versus untreated controls; ^^^^p < 0.0001 versus the PA-only group (one-way ANOVA with Tukey’s post-hoc test).

### Modulation of endothelial adhesion molecule expression

Given the central role of vascular cell adhesion molecule-1 and intercellular adhesion molecule-1 in recruiting circulating monocytes to activated endothelium, we subsequently assessed whether GPR84 activation modulates expression of these critical adhesion receptors. Palmitic acid challenge markedly induced VCAM-1 mRNA expression above baseline levels, whereas coincubation with 6-OAU or ZQ-16 attenuated this transcriptional response in a statistically significant manner (Figure 3A). Parallel experimental conditions demonstrated comparable reductions in ICAM-1 transcript levels following agonist treatment (Figure 3B). Protein quantification via specific enzyme-linked immunosorbent assays confirmed that agonist exposure significantly decreased VCAM-1 secretion into culture supernatants (Figure 3C), while ICAM-1 protein concentrations showed analogous reductions under identical treatment conditions (Figure 3D). Taken together, these results reveal that GPR84 activation dampens endothelial activation phenotypes by downregulating key adhesion receptors required for leukocyte attachment.

**Figure 3:** – Agonist treatment reduces endothelial adhesion molecule expression. Following 24-hour exposure to 1 mM PA in the presence or absence of the indicated agonists (1 μM each), human aortic endothelial cells (HAECs) were assessed for vascular cell adhesion molecule-1 (VCAM-1) and intercellular adhesion molecule-1 (ICAM-1) expression. (A, B) mRNA levels determined by qPCR. (C, D) Secreted protein concentrations measured by enzyme-linked immunosorbent assay (ELISA). ****p < 0.0001 versus untreated controls; ^^^^p < 0.0001 versus the PA-only group (one-way ANOVA with Tukey’s post-hoc test).

### Functional inhibition of monocyte-endothelial adhesion

To determine whether the observed molecular alterations culminate in functional changes to cellular interactions, we evaluated THP-1 monocyte adhesion to treated endothelial monolayers using quantitative fluorescence methodologies. Microscopic examination of calcein-AM-stained monocytes revealed dense punctate adherence to palmitic acid-treated HAEC monolayers, whereas cultures receiving coincubation with GPR84 agonists displayed markedly attenuated fluorescence intensity indicating reduced cellular attachment (Figure 4A). Quantitative image analysis substantiated these qualitative observations, demonstrating that both 6-OAU and ZQ-16 reduced monocyte attachment indices by approximately fifty percent relative to palmitic acid stimulation alone (Figure 4B). Such functional evidence indicates that GPR84 agonism disrupts the adhesive interface between activated endothelial cells and circulating monocytes under pathophysiologically relevant conditions.

**Figure 4:** – GPR84 activation inhibits monocyte adhesion to endothelial monolayers. Human aortic endothelial cells (HAECs) pretreated for 24 hours with 1 mM PA ± GPR84 agonists were co-cultured for 1 hour with calcein-acetoxymethyl ester (calcein-AM)-loaded THP-1 monocytes. (A) Representative fluorescence micrographs showing adherent cells (scale bar, 50 μm). (B) Quantification of bound monocytes normalized to untreated controls. ****p < 0.0001 versus untreated controls; ^^^^p < 0.0001 versus the PA-only group (one-way ANOVA with Tukey’s post-hoc test).

### Suppression of NF-κB signaling pathways

We proceeded to investigate the intracellular signaling mechanisms governing these anti-inflammatory effects, specifically focusing upon the nuclear factor-κB transcriptional pathway, given its established role in regulating inflammatory gene expression. Western blot analysis of nuclear protein extracts demonstrated that palmitic acid treatment promoted significant nuclear translocation of the NF-κB p65 subunit, and this effect was substantially diminished in cells receiving concurrent GPR84 agonist treatment (Figure 5A). Luciferase reporter assays utilizing NF-κB-specific promoter constructs further confirmed that agonist treatment markedly suppressed NF-κB transcriptional activity compared with palmitic acid stimulation alone (Figure 5B). These mechanistic observations imply that GPR84 signaling interferes with canonical NF-κB activation pathways, thereby attenuating downstream inflammatory gene transcription.

**Figure 5:** – GPR84 activation suppresses NF-κB (nuclear factor-kappa B) activation. Human aortic endothelial cells (HAECs) were stimulated for 24 hours with 1 mM palmitic acid (PA), either alone or together with 1 μM 6-n-octylaminouracil (6-OAU) or 1 μM ZQ-16. (A) Nuclear fractions were immunoblotted for p65; Lamin B1 served as a loading control. (B) NF-κB-driven luciferase activity normalized to Renilla control. ****p < 0.0001 compared with untreated controls; ^^^^p < 0.0001 versus the PA-only group (one-way ANOVA with Tukey’s post-hoc test).

### Preservation of gfi1 expression by GPR84 activation

We hypothesized that the transcriptional repressor Gfi1 might participate in GPR84-mediated cytoprotection through previously characterized interactions with NF-κB signaling components. Palmitic acid exposure significantly decreased Gfi1 mRNA abundance compared with untreated controls, whereas concurrent treatment with either 6-OAU or ZQ-16 maintained expression levels comparable to baseline (Figure 6A). Immunoblot analysis utilizing Gfi1-specific antibodies validated that protein levels followed similar expression patterns, with agonist pretreatment effectively preventing palmitic acid-induced Gfi1 depletion (Figure 6B). These observations support the premise that GPR84 activation preserves Gfi1 expression under inflammatory conditions, potentially maintaining transcriptional repression of pro-inflammatory target genes.

**Figure 6:** – GPR84 signaling maintains growth factor independence 1 (Gfi1) expression under inflammatory conditions. Human aortic endothelial cells (HAECs) were incubated for 24 hours with 1 mM PA ± GPR84 agonists. (A) Gfi1 mRNA quantified by qPCR and normalized to 18S rRNA. (B) Representative immunoblots and densitometric analysis of Gfi1 protein relative to β-actin. ****p < 0.0001 compared with untreated controls; ^^^^p < 0.0001 versus the PA-only group (one-way ANOVA with Tukey’s post-hoc test).

### Requirement of Gfi1 for GPR84-mediated protective effects

To establish definitively whether Gfi1 is required for agonist efficacy rather than merely associated with protective responses, we employed RNA interference to deplete Gfi1 before pharmacological stimulation with receptor agonists. Adeno-viral delivery of Gfi1-specific short hairpin RNA achieved efficient protein knockdown, reducing Gfi1 immunoreactivity by approximately seventy percent compared with scrambled control sequences (Figure 7A). Under these knockdown conditions, the capacity of 6-OAU and ZQ-16 to suppress NF-κB transcriptional activity was abolished (Figure 7B). Similarly, Gfi1 depletion reversed agonist-mediated reductions in VCAM-1 protein secretion (Figure 7C) and ICAM-1 protein production (Figure 7D). Functional adhesion assays subsequently confirmed that without intact Gfi1 expression, GPR84 agonists failed to attenuate monocyte attachment to endothelial monolayers (Figure 7E). Collectively, these rescue experiments confirm that Gfi1 is indispensable for GPR84-mediated anti-inflammatory and anti-adhesive effects, establishing this transcription factor as a critical proximal mediator in the signaling cascade.

**Figure 7:** – Gfi1 depletion reverses agonist-mediated protection. Human aortic endothelial cells (HAECs) were transduced for 48 hours with adenoviruses encoding Gfi1-targeting short hairpin RNA (shRNA) or scrambled control shRNA, then stimulated for 24 hours with 1 mM palmitic acid (PA) ± 1 μM GPR84 agonists (6-OAU or ZQ-16). (A) Representative immunoblot and quantification confirming efficient Gfi1 knockdown (****P < 0.0001 compared with scrambled control shRNA group). (B) Nuclear factor-κB (NF-κB) luciferase reporter activity normalized to Renilla control. (C, D) Secreted vascular cell adhesion molecule-1 (VCAM-1) and intercellular adhesion molecule-1 (ICAM-1) protein levels measured by enzyme-linked immunosorbent assay (ELISA). (E) Quantification of calcein-AM-labeled THP-1 monocyte adhesion. For panels B-E: ****P < 0.0001 compared with the untreated control group; ^^^^P < 0.0001 compared with the PA-only group; ††P < 0.01 compared with the corresponding PA + agonist group (two-way ANOVA with Šidák’s post-hoc test).

## Discussion

The present investigation delineates a cytoprotective signaling cascade wherein GPR84 activation attenuates palmitic acid-induced endothelial inflammation via preservation of Gfi1 expression and subsequent inhibition of nuclear factor-κB transcriptional activity.^23^ These observations diverge substantively from prior characterizations depicting GPR84 as a predominantly pro-inflammatory receptor within myeloid lineages, thereby establishing context-dependent functional diversity that necessitates careful mechanistic dissection.^18^

The Central Figure is a Schematic illustration of the protective mechanism of GPR84 agonism against FFA-induced endothelial inflammation. GPR84 activation preserves Gfi1 expression, which in turn inhibits NF-κB p65 nuclear translocation, leading to reduced expression of pro-inflammatory cytokines (IL-1β, TNF-α, MCP-1) and adhesion molecules (VCAM-1, ICAM-1), ultimately attenuating monocyte adhesion to the endothelium.

**Central Illustration:** : Agonism of GPR84 Attenuates Free Fatty Acid-Induced Endothelial Inflammation and Monocyte Adhesion via Gfi1/NF-κB Pathway

Previous investigations demonstrated that Nesfatin-1 protects HAECs against free fatty acid-induced inflammation through identical Gfi1-dependent suppression of nuclear factor-kappa B signaling.^23^ The current study extends this mechanistic paradigm by identifying GPR84 as an upstream pharmacological trigger capable of activating this protective pathway. While both investigations utilize identical cellular models and inflammatory stimuli, the present work uniquely identifies a druggable receptor target responding to synthetic agonists with favorable pharmaceutical properties.^23^

The anti-inflammatory effects of GPR84 agonism observed in endothelial cells extend beyond the canonical pro-inflammatory functions previously ascribed to this receptor in myeloid cells, underscoring the importance of cellular context in determining GPR84 signaling outcomes^24^. Recio et al. established that GPR84 agonism enhances cytokine secretion, chemotaxis, and phagocytic activity in macrophages under inflammatory conditions, suggesting the receptor amplifies innate immune responses.^24^ Similarly, Nicol et al. demonstrated that GPR84 mediates mechanical hypersensitivity and thermal hypersensitivity in neuropathic pain models through peripheral macrophage activation.^17^ These pro-inflammatory actions have motivated the development of GPR84 antagonists for ulcerative colitis, fibrotic diseases, and neuropathic pain.^25^ However, recent investigations reveal that GPR84 function varies significantly depending on cellular context and metabolic environment.^26^

The current findings align with emerging evidence that GPR84 functions as an immunomodulating receptor rather than a simple pro-inflammatory mediator.^26^ Under lipotoxic conditions characterized by excessive saturated fatty acid exposure, GPR84 activation appears to suppress rather than amplify inflammatory signaling, potentially representing a compensatory homeostatic mechanism.^23^ This context-dependent functionality may explain apparent contradictions between the present endothelial findings and prior macrophage studies, as the metabolic milieu and cellular differentiation state likely determine downstream signaling outcomes.^26^

The identification of Gfi1 as a critical downstream mediator of GPR84 cytoprotection constitutes a significant mechanistic advance.^27^ Previous investigations established Gfi1 as a zinc finger transcriptional repressor that antagonizes nuclear factor-kappa B p65 DNA binding, thereby limiting inflammatory cytokine production.^27^ However, these prior studies focused predominantly on hematopoietic lineages, leaving the functional role of Gfi1 in endothelial cells under metabolic stress largely unexplored.^28^ The present demonstration that Gfi1 knockdown reverses GPR84-mediated protection confirms that this transcription factor operates as a non-redundant signaling node within vascular endothelium.^27^

Several limitations constrain the interpretation of these findings. The investigation relied exclusively upon in vitro cell culture models utilizing static two-dimensional monolayers, which lack the physiological hemodynamic shear stress, multicellular complexity, and three-dimensional architecture characteristic of arterial vasculature.^28^ Conventional tissue culture plastic models demonstrate poor physiological relevance for atherosclerosis research, as they cannot replicate the early stages of plaque formation, endothelial permeability dynamics, or interactions with circulating blood components.^29^ Future investigations should employ microfluidic organ-on-chip technologies that mimic vascular shear flow and enable co-culture with smooth muscle cells and monocytes under dynamic conditions.^29^ Furthermore, our experiments were confined to HAECs. Verification of the GPR84–Gfi1–NF-κB axis in other endothelial subtypes, such as human umbilical vein endothelial cells (HUVECs) or human coronary artery endothelial cells (HCAECs), will be necessary to establish broader vascular relevance.

Additionally, the study utilized supraphysiological concentrations of palmitic acid that may induce non-specific cytotoxicity rather than selective inflammatory activation, potentially limiting translational relevance.^25^ The absence of in vivo validation represents another critical constraint, as atherogenesis involves complex multicellular interactions between endothelium, circulating leukocytes, and smooth vascular muscle cells that cannot be recapitulated in monoculture systems.^25^ Animal studies utilizing high-fat diet-fed models with endothelial-specific GPR84 manipulation would provide essential confirmation of the protective mechanism observed in vitro.^17^ In addition, only two synthetic GPR84 agonists (6-OAU and ZQ-16) were examined; validation with a broader panel of structurally distinct agonists would reinforce the generalizability of the protective effects and help rule out potential off-target activities.

Future investigations should explore the precise molecular linkage between GPR84 receptor activation and Gfi1 transcriptional upregulation.^30^ Potential mechanisms may involve Gi/o-mediated inhibition of adenylate cyclase, subsequent modulation of protein kinase A activity, and alterations in transcription factor phosphorylation states that enhance Gfi1 promoter accessibility.^30^ Additionally, the therapeutic potential of GPR84 agonists for vascular protection in metabolic disorders requires evaluation in preclinical models of atherosclerosis, with particular attention to potential divergent effects on endothelial versus myeloid cell populations.^23^ The development of tissue-specific delivery systems or biased agonists that selectively activate endothelial protective pathways while minimizing macrophage pro-inflammatory responses may optimize therapeutic indices.^26^

## Conclusion

In summary, these findings establish GPR84 as a context-dependent modulator of endothelial inflammation that operates through a Gfi1-dependent mechanism distinct from its characterized roles in immune cells. This investigation expands understanding of metabolic sensing in vascular endothelium and identifies a novel receptor-transcription factor axis with potential therapeutic implications for atherosclerotic vascular disease.
